# Antimicrobial Activities of Polyethylene Terephthalate-Waste-Derived Nanofibrous Membranes Decorated with Green Synthesized Ag Nanoparticles

**DOI:** 10.3390/molecules28145439

**Published:** 2023-07-16

**Authors:** Tannaz Soltanolzakerin-Sorkhabi, Mehrab Fallahi-Samberan, Vignesh Kumaravel

**Affiliations:** 1 Department of Chemical Engineering, Ahar Branch, Islamic Azad University, Ahar P.O. Box 5451116714, Iran; tannaz.soltanolzakerin-sorkhabi@dokt.p.lodz.pl; 2 International Centre for Research on Innovative Biobased Materials (ICRI-BioM)-International Research Agenda, Lodz University of Technology, Zeromskiego 116, 90-924 Lodz, Poland

**Keywords:** membranes, nanofibers, antibacterial, green synthesis, waste recycling

## Abstract

Thermoplastic polymers are one of the synthetic materials produced with high tonnage in the world and are so omnipresent in industries and everyday life. One of the most important polymeric wastes is polyethylene terephthalate (PET), and the disposal of used PET bottles is an unsolved environmental problem, and many efforts have been made to find practical solutions to solve it. In this present work, nanofibrous membranes were produced from waste PET bottles using the electrospinning process. The surface of membranes was modified using NaOH and then decorated with green synthesized Ag nanoparticles (10 ± 2 nm) using an in situ chemical reduction method. The morphology, size, and diameter of the Ag nanoparticles decorating the nanofibers were characterized through transmission electron microscopy (TEM), a field emission scanning electron microscope (FESEM), Fourier transform infrared spectroscopy (FTIR), X-ray diffraction (XRD), and UV-visible spectroscopy techniques. Finally, the antimicrobial activity of the nanofibrous membranes was tested against *Escherichia coli*, *Pseudomonas aeruginosa*, and *Staphylococcus aureus* using disc diffusion and colony-forming count methods. The growth of bacteria was not affected by the pure nanofibrous membranes, while the Ag-decorated samples showed inhibition zones of 17 ± 1, 16 ± 1, and 14 ± 1 mm for *Pseudomonas aeruginosa*, *Escherichia coli*, and *Staphylococcus aureus*, respectively. The planktonic culture results of *Pseudomonas aeruginosa* showed that the membranes had a relatively low inhibitory effect on its growth. The obtained results showed that *Pseudomonas aeruginosa* has a relatively low ability to form biofilms on the nanostructured membranes too. A good agreement was observed between the data of biofilm formation and the planktonic cultures of bacteria. The plastic-waste-derived PET/Ag nanocomposite membranes can be used for wound dressings, air filters, and water purification applications.

## 1. Introduction

Polyethylene terephthalate (PET) from the polyester family is one of the most commonly used thermoplastics for engineering materials, fibers, and textiles, and it has many uses in the packaging industry in various forms, including beverage containers. The global consumption of PET bottles is increasing every day, and at the moment, it has reached around 481.6 billion per year. Some characteristics of PET bottles, such as resistance against gas diffusion breakage and thermal stability, make them a nonbiodegradable material and a significant polymer waste; therefore, it has adverse and damaging effects on the environment. Nowadays, the material is responsible for 8 percent by weight and 12 percent by volume of solid waste in the world, so recycling PET waste is very important for the conservation of resources and the protection of the environment. PET recycling not only helps to solve the problem of solid waste but also as a source of raw material for some industries. Furthermore, the economic and best solution to solve the problem of PET waste is the recycling process [1,2,3,4,5,6,7]. With the advent and rapid growth of nanotechnology and nanoscience, a relatively simple and versatile technique called electrospinning has been introduced to produce polymer nanofibers. The electrospinning process is able to produce nanostructures with a very large specific surface area in the forms of nanofibers and nanofibrous membranes, with excellent properties, including chemical, physical, and mechanical properties [8,9,10]. Electrospinning offers many advantages, such as control over morphology, porosity, and composition [11], and it is a relatively simple and low-cost method to generate polymer nanofibers [12]. Up to now, several electrospun nanofibers have been fabricated from a wide variety of polymeric materials, including a polymer/inorganic nanocomposite based on both natural and synthetic polymers [13,14,15]. Nanosensors [16,17], controlled drug delivery systems [18,19,20,21,22], wound healing [23,24,25], tissue engineering [26,27,28], energy devices [29], filtration [30,31], distillation [32,33], and the determination of environmental pollutants and photocatalysts [34,35] are some of the applications of electrospun nanofibers and nanofibrous membranes. Today, electrospun nanofibers incorporated with antibacterial agents are widely used for antimicrobial applications due to their enhanced antimicrobial performance compared to traditional antimicrobial materials [36]. This excellent property is provided by the encapsulation or attachments of antibacterial agents, such as metal or metal-oxide nanoparticles and antibiotics, onto or into the supporting nanofibers [37].

Nanostructured Ag is considered a new generation of antibacterial agent, with great potential for use in various antibacterial surfaces for medical devices, food packages, and so on [38,39,40,41,42,43,44,45]. According to the recent research, nanostructured Ag potentiates the antimicrobial activity of gentamicin, tetracycline, ampicillin, ofloxacin, and chloramphenicol against *Escherichia coli* in vitro and in animal models [46,47], tobramycin against biofilm-producing *Escherichia coli* and *Pseudomonas aeruginosa* [44], and vancomycin against *Escherichia coli* [48]. Due to very small dimensions in the nanoscale, electrospun polymer nanofibers are an ideal carrier and substrate for the immobilization of Ag nanoparticles. The electrospun carrier can cause nanoparticles to distribute homogeneously and prevent their mass aggregation. In other words, the applicability of Ag nanoparticles on the electrospun polymer nanofibers can be increased [49,50,51,52,53,54].

Turmeric powder is composed of 60–70% carbohydrates, 6–13% water, 6–8% protein, 5–10% fat, 3–7% dietary minerals, 2–7% dietary fiber, 3–7% essential oils, and 1–6% curcumin [55]. Curcumin with its hydroxyl groups can reduce metal salts into their respective metal nanoparticles [56]. The reducing and stabilizing role of turmeric in the synthesis of Ag nanoparticles has been investigated, and Ag nanoparticle formation was indicated by the color changes of the turmeric/Ag^+^ ions’ aqueous solution from orange to dark yellow/ yellowish green [57]. The reduction of Ag^+^ to Ag^0^ has been studied using turmeric as a reducing agent in the presence of NaOH [57]. It has been reported that the reaction of turmeric accelerated significantly in the basic media. Ag nanoparticles have been synthesized using tea, garlic, and turmeric extracts, and the difference between the obtained Ag nanoparticles has been evaluated [58]. According to the reported results, Ag nanoparticles synthesized using turmeric were smaller in size compared to the ones prepared using tea and garlic extracts. Some researchers [59,60] were able to synthesize Ag nanoparticles through a green solid-state reaction using turmeric and Ag nitrate. According to their reports, turmeric plays a substantial role in the stabilization and formation of uniform Ag nanoparticles. However, according to our knowledge, the in situ green synthesis of Ag nanoparticles on electrospun nanofibrous membranes based on waste PET has not reported for antimicrobial application up to now. This study aimed to fabricate antibacterial waste PET/Ag membranes. First, the nanofibrous PET membrane was prepared using electrospinning and then carboxylated using NaOH. The obtained sample was decorated with Ag nanoparticles using in situ green synthesis. Turmeric extract was used as a green reducing agent. The antibacterial activities of synthesized electrospun membranes were evaluated against three important Gram-negative and Gram-positive bacteria, *Escherichia coli*, *Pseudomonas aeruginosa*, and *Staphylococcus aureus*. The objective of this research was to lower the amount of environmental waste that PET usage generates and convert it into a valuable product that could be used in biomedical applications.

## 2. Results and Discussion

### 2.1. Characterization

**Figure 1:**
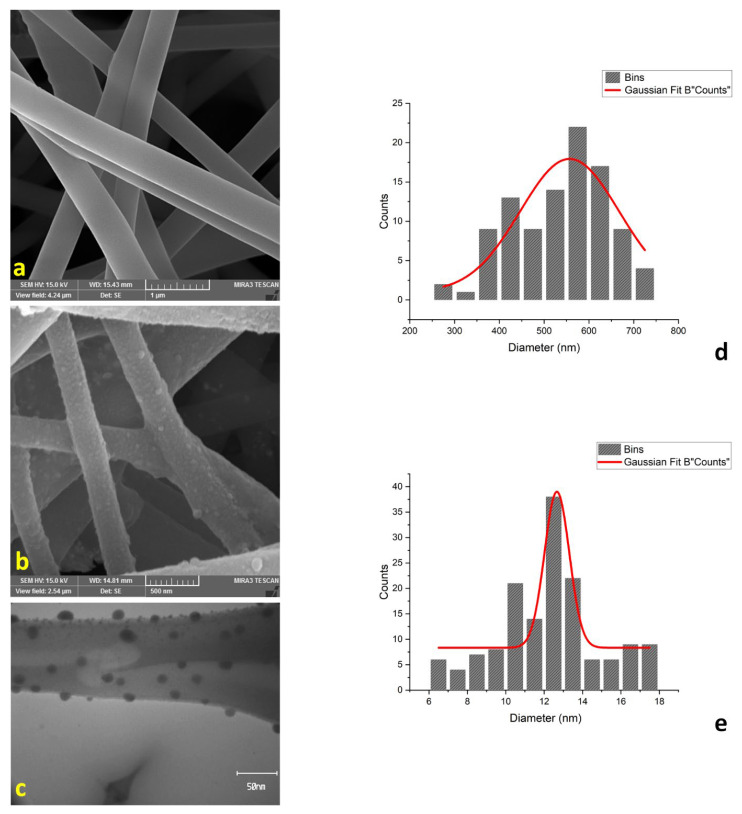
(**a**) FE-SEM image of pristine PET membrane, (**b**) FE-SEM image of PET/Ag membrane, (**c**) TEM image of PET/Ag membrane, (**d**) nanofibers’ size distribution, and (**e**) Ag nanoparticles’ size distribution.

The FE-SEM and TEM results of pure and PET/Ag electrospun membranes are shown in Figure 1. The pristine electrospun PET membrane has a continuous and uniform nanofiber with a smooth and bead-free surface (Figure 1a). The average diameter of the electrospun nanofibers is 560 ± 20 nm (Figure 1d). Also, the energy dispersive spectroscopy (EDS) of the pure and PET/Ag electrospun membranes are shown in Table 1 and Table 2, respectively. The presence of elemental carbon in the EDS was from the chemical structure of PET, and elemental oxygen was due to the carbonyl groups in the PET structure. The TEM image (Figure 1c) revealed that uniformly distributed Ag nanoparticles are successfully formed via the in situ reduction of Ag nitrate by turmeric (reducing agent) and are homogeneously immobilized on the electrospun PET nanofibers. The average particle size of Ag nanoparticles on the PET nanofiber surface was in the range of 7–16 nm, with an average diameter of 10 ± 2 nm (Figure 1e). The obtained PET/Ag membrane (Figure 1b) can be effectively used for antibacterial applications.

**Figure 2:**
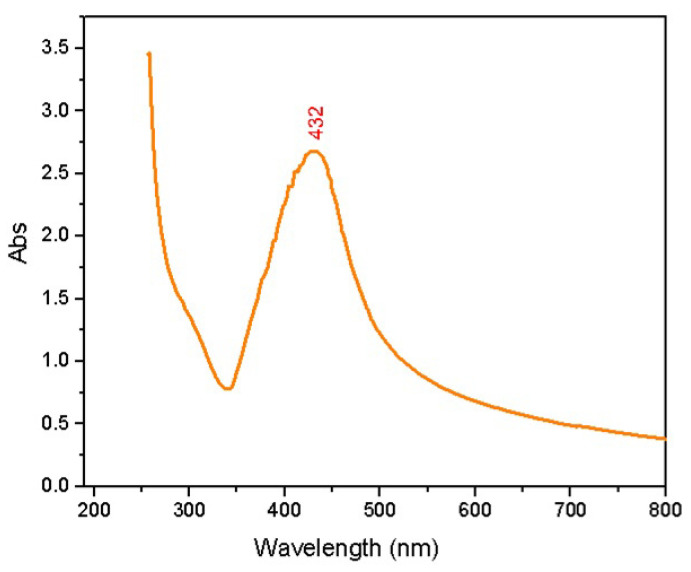
UV-visible spectrum of electrospun PET/Ag membrane.

**Scheme 1:**
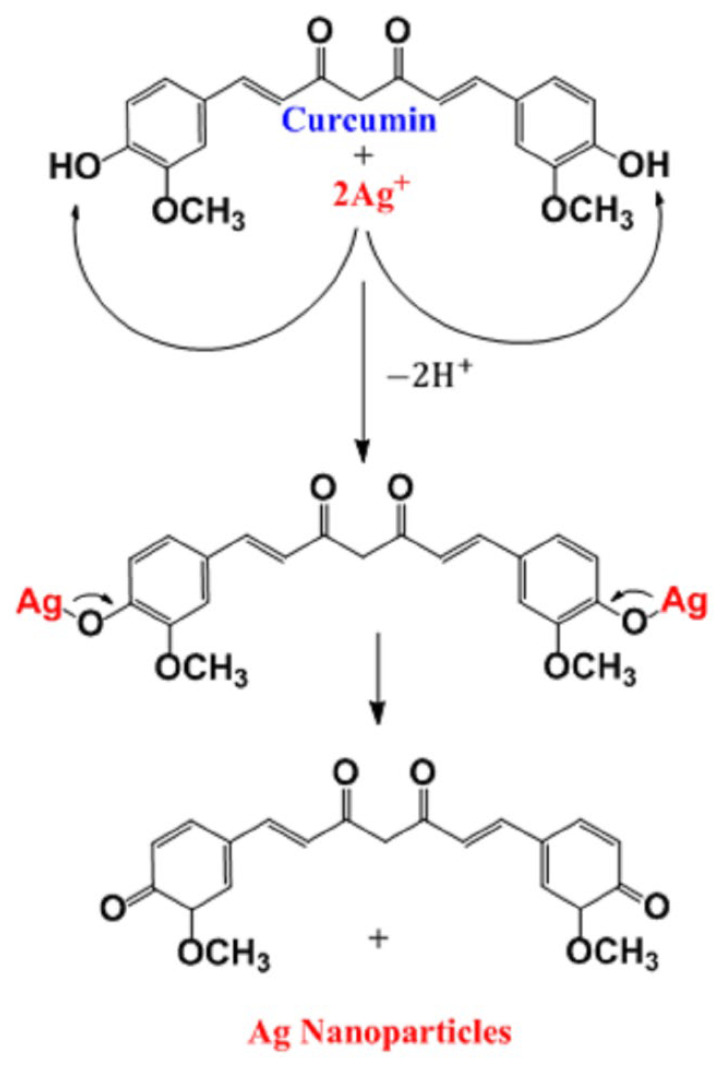
Schematic of the proposed mechanism for Ag nanoparticle formation.

**Scheme 2:**
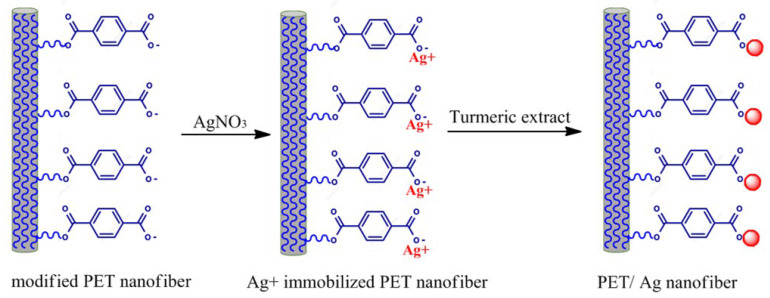
Schematic of PET/Ag membrane fabrication.

The main component (60–70%) of the turmeric extract is curcuminoids, a natural phenolic compound belonging to the ginger family (Zingiberaceae). The turmeric extract also contains small amounts of alkaloids, triterpenoids, and sterols [61]. The main component of curcuminoids is curcumin, a light-yellow compound. Some of the applications of curcumin are in the food industry, traditional medicines, and coloring agents. Curcumin in the turmeric extract shows strong antioxidant activity comparable to vitamins C and E. It is the antioxidant property of the turmeric extract that reduces the Ag^+^ cations in the green synthesis of the Ag nanoparticles. The modification of the electrospun PET nanofibers using NaOH and the formation of Ag nanoparticles on the nanofibers are presented in Scheme 1 and Scheme 2, respectively. NaOH created carboxylic groups in the modification process on the PET nanofiber surface. The carboxylic groups attracted Ag^+^ ions from the AgNO_3_ solution and entrapped them. The Ag ions were converted into Ag nanoparticles in the reduction process, and an electrospun PET/Ag membrane was made. Figure 2b shows that NaOH cannot affect the PET nanofiber size and morphology, especially in the short treatment time (5 min) and at a relative low temperature (60 °C). It is worth mentioning that the so-called topochemical reaction occurred between the PET nanofibers and NaOH, and hence, it was confined to the surface of the nanofibers [62].

When the carboxylated PET membrane was immersed in the AgNO_3_ solution (pH 7), some -COOH groups changed into -COO^−^ anions. The Ag^+^ cations were absorbed and immobilized around the -COO^−^ anions through the strong electrostatic force. The antioxidants in the turmeric extract reduced the Ag ions to form Ag nanoparticles on the PET nanofibers of the membrane. The schematic of the proposed mechanism for Ag nanoparticle formation in the presence of curcumin is shown in Scheme 1, and different methods of Ag cluster size growth are presented in Equations (1)–(5) [63]. In addition, the formation of the PET/Ag membrane is illustrated in Scheme 2.


(1) $${Ag}^{0}+{Ag}^{0}\;\to \;{Ag}_{2}$$

(2) $${Ag}^{0}+{Ag}^{+}\;\to \;({Ag}_{2}{)}^{+}$$

(3) $${Ag}_{n}+{Ag}^{+}\;\to \;({Ag}_{n+1}{)}^{+}$$

(4) $$({Ag}_{n}{)}^{+}+{Ag}^{0}\;\to \;({Ag}_{n+1}{)}^{+}$$

(5) $${Ag}_{n}+{Ag}^{0}\;\to \;({Ag}_{n+1})$$

The UV-visible spectrum of the Ag nanoparticles grown on the nanofibers of the chemically modified PET membrane is shown in Figure 2. The appearance of an absorption band with a sharp maximum at 432 nm indicates very well the spectral behavior of Ag nanoparticles [39]. The existence of the discrete energy levels of electrons in the Ag nanoparticles resulted in high optical absorption [64]. A relatively high absorption band with a maximum of about 400 nm is observable in the Ag nanoparticles with diameters less than 5 nm. The absorption band broadened with the increasing size of the Ag nanoparticles and shifted to higher wavelengths [65]. As a result, the formation of spherical Ag nanoparticles on the surface of the PET nanofibers was confirmed using maximal absorbance at 432 nm in the UV spectrum of the PET/Ag membrane.

**Figure 3:**
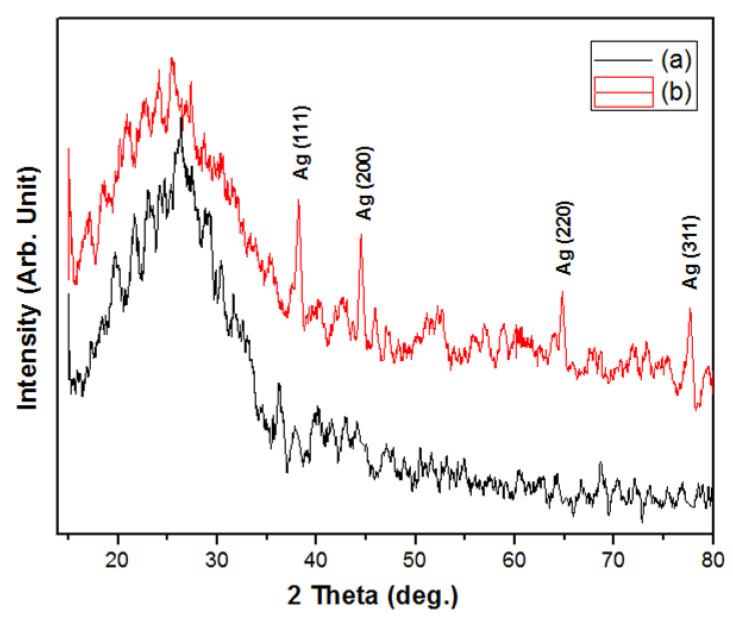
XRD patterns of (**a**) PET and (**b**) PET/Ag membranes.

Figure 3 shows the XRD patterns of the pristine and PET/Ag membranes. A broad peak at a 2θ value of about 26 was observed for pristine PET. For the PET/Ag samples, four additional peaks were seen in the face-center-cubic Ag phase. The peaks appeared at 38.2°, 44.5°, 64.8°, and 77.7° and are attributed to the (111), (200), (220), and (311) planes of Ag (ICCD 4-783) [57]. The XRD results also demonstrate that the silver nanoparticles on the PET membrane are crystalline. Only metallic nanostructured Ag was synthesized using turmeric extract reduction due to the absence of the characteristic peaks of silver oxide.

**Figure 4:**
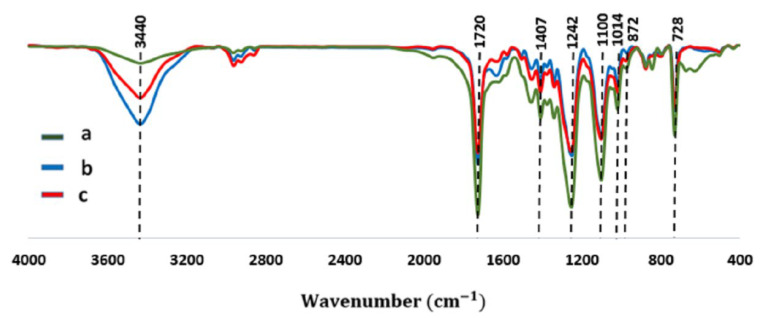
FTIR spectra of (**a**) pristine, (**b**) modified, and (**c**) PET/Ag membranes.

The FT-IR spectra of pristine, modified PET, and PET/Ag electrospun membranes are shown in Figure 4. The absorption peaks at 3440 cm^−1^ and 2990 cm^−1^ are attributed to the stretching vibration of the hydroxyl groups and the asymmetric vibration of the C–H bonds [66]. The peaks for the stretching of C=O and the stretching of ring C–C in the PET structure are observed at 1720 cm^−1^ [67] and 1407 cm^−1^ [67,68]. The peaks seen at 1242 cm^−1^ and 1100 cm^−1^ correspond to the stretching of the (C=O)–C and C–O stretch bonds [67,68]. The absorbance at 1100 cm^−1^ is due to O–CH_2_ stretching, and the peak at 1014 cm^−1^ is due to the in-plane deformation of ring C–H. Also, the bands at 872 cm^−1^ [68,69] and 728 cm^−1^ are attributed to the out-of-plane vibration ring C–H and ring C–C bending [67,68]. These peaks also appeared in the modified electrospun PET membrane and electrospun PET/Ag membrane. The main difference between the FTIR spectra of the pristine and modified electrospun PET membranes is the absorbance intensity of O–H at 3440 cm^−1^. The reason for this is that more OH groups formed after the surface modification of the PET nanofibers by NaOH. On the other hand, the absorbance intensity of the OH reduced in the spectrum of the PET/Ag membrane compared to the modified membrane. Also, some characteristic absorption peaks of the samples containing Ag slightly shifted compared to the pristine PET. These results confirm the surface modification and decoration of Ag nanoparticles for PET nanofibers.

### 2.2. Antimicrobial Activity

**Figure 5:**
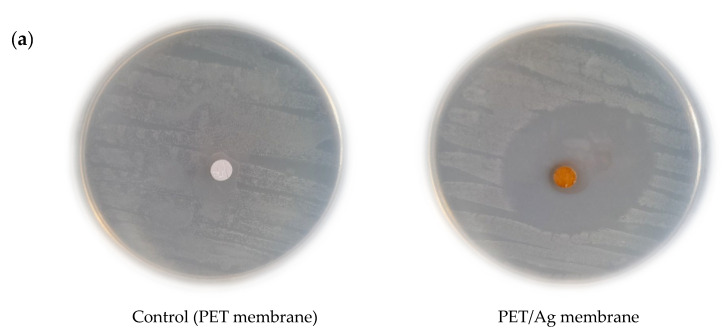

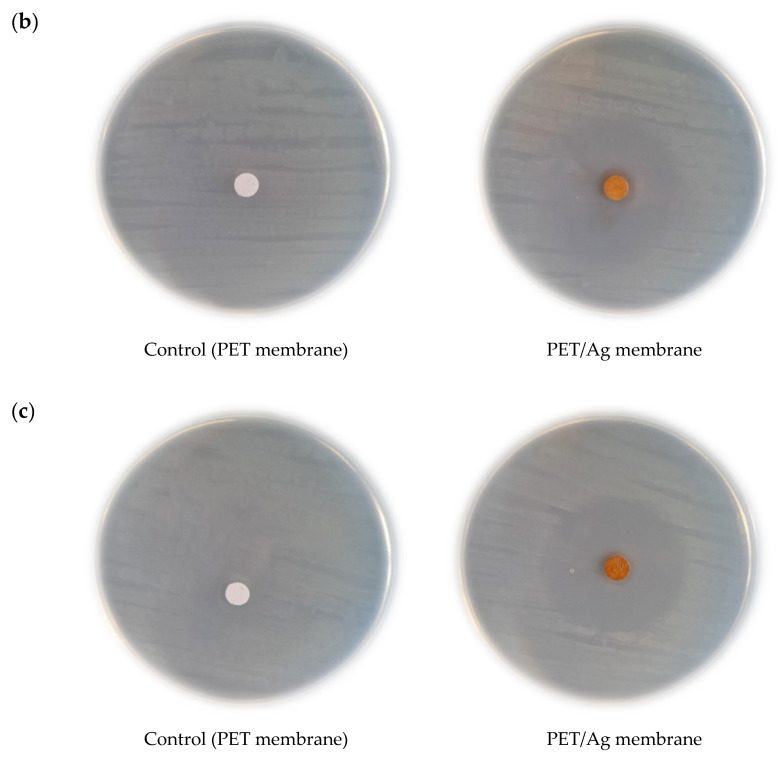
The results of antimicrobial activity of the PET/Ag membrane on agar plates against (**a**) *Escherichia coli*, (**b**) *Pseudomonas aeruginosa* and (**c**) *Staphylococcus aureus*.

The antibacterial activities of the PET/Ag membrane were examined against *Staphylococcus aureus*, *Pseudomonas aeruginosa*, and *Escherichia coli*. The bacteria attachment was not affected by the pristine membranes on the agar plate, while the PET/Ag membrane showed inhibition zones of 17 ± 1, 16 ± 1, and 14 ± 1 mm for *Pseudomonas aeruginosa*, *Escherichia coli*, and *Staphylococcus aureus*, respectively (Figure 5).

Undesirable microorganisms, such as pollutants, have many adverse effects on the environment in various fields and especially have many negative consequences for human health. Most microorganisms tend to adhere to different surfaces and interfaces, although, they can grow planktonically. Removing microorganisms that are adhered to solid surfaces is more difficult than removing microorganisms grown in a liquid media (planktonic growth) due to the formation of biofilms. In biofilms, cells are more resistant to various stressors and have different biochemical and genetic behavior from planktonic ones. Nowadays, many alternatives are being investigated to reduce microbial colonization on biomaterials, raw materials, industrial equipment, and medical instruments.

**Figure 6:**
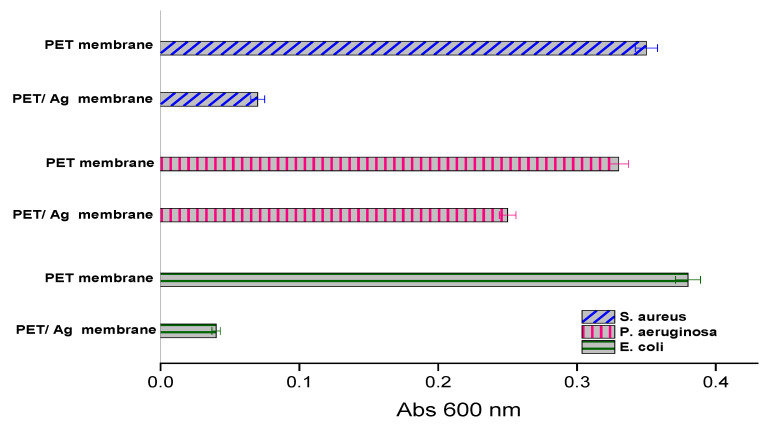
Turbidity of the microbial suspension (absorbance) determined using UV–visible spectroscopy at 600 nm after cultivation for 24 h in PET and PET/Ag membranes.

The inhibitory effect of the PET/Ag membrane on the planktonic growth of microbes was investigated after an incubation time of 24 h. The recorded absorbance values of *Staphylococcus aureus*, *Pseudomonas aeruginosa*, and *Escherichia coli* cultures are presented in Figure 6. As the turbidity of the microbial suspension indicates, the PET/Ag electrospun membrane had the highest effect against the growth of *Escherichia coli* in the liquid and then on the growth (cell development and division) of *Staphylococcus aureus* compared to the control (the PET membrane). The relatively low inhibitory effect of this sample belonged to the planktonic cultures of *Pseudomonas aeruginosa*.

**Figure 7:**
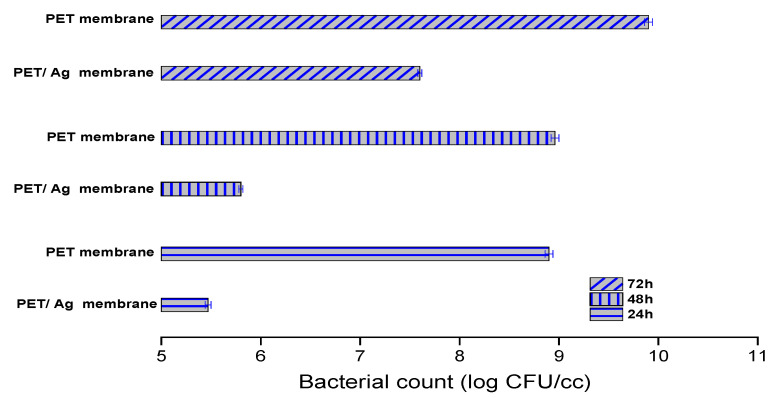
The number of living cells of *Staphylococcus aureus* after cultivation for 24 h, 48 h, and 72 h at 37 °C.

**Figure 8:**
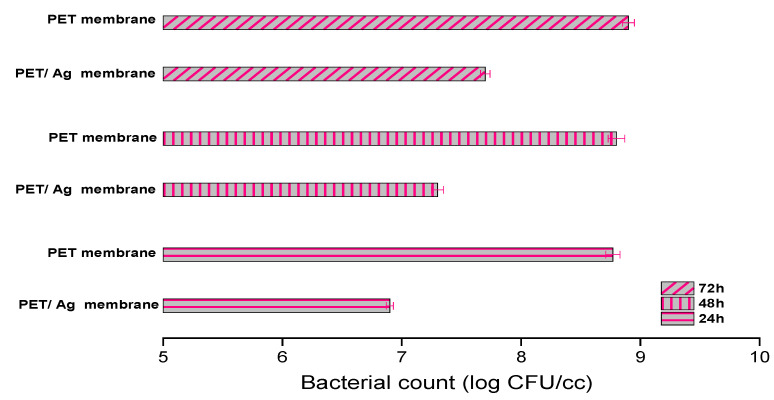
The number living cells of *Pseudomonas aeruginosa* viable cells after cultivation for 24 h, 48 h, and 72 h at 37 °C.

**Figure 9:**
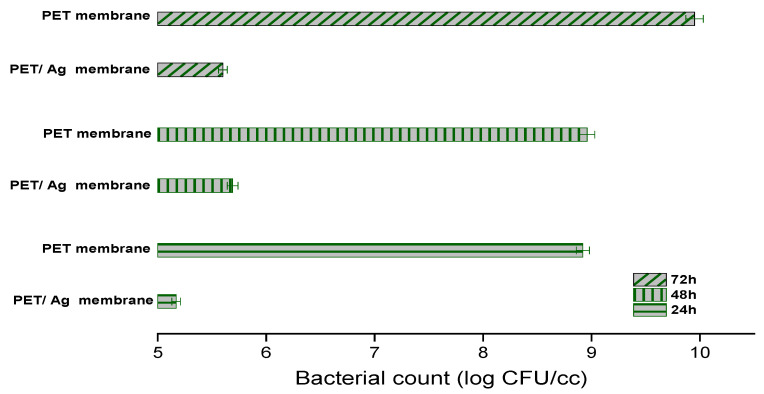
The number of living cells of *Escherichia coli* viable cells after cultivation for 24 h, 48 h, and 72 h at 37 °C.

After cultivation for another 24 h, 48 h, and 72 h at 37 °C, a monolayer biofilm formed and grew on the surface of the nanofibrous membranes. The number of living cells of *Staphylococcus aureus*, *Pseudomonas aeruginosa*, and *Escherichia coli* forming the biofilm are presented as log colony-forming units (CFU)/cc in Figure 7, Figure 8 and Figure 9.

The evaluation of biofilm formation and its development potential has shown similar results to those obtained in the liquid media. The reduction in the living cells due to the presence of a bactericidal agent (Ag nanoparticles) on the surface of the PET membrane has resulted in an antibiofilm effect. According to the obtained results, the PET/Ag membrane has efficiently inhibited the formation and development of biofilm on the surface compared to the pristine PET membrane. The efficiency of the inhibition of film formation decreased with incubation time, and this inverse trend was observed for all three bacteria. From the results shown in Figure 6, Figure 7 and Figure 8, it is obvious that all stages of the biofilm development, from the initial period (up to 24 h) to the completion of the biofilm (up to 72 h), were affected by the presence of the bactericidal agent on the PET/Ag membrane for all three bacteria. In the case of *Pseudomonas aeruginosa*, it should be noted that it can efficiently colonize and readily grow in various environments due to it is intrinsic persistence and tolerance. *Pseudomonas aeruginosa* biofilms are very hard to destroy with current bactericidal agents. Compared to the other microbes, the potential of biofilm formation and development on the fabricated membranes was low for *Pseudomonas aeruginosa*. In general, the antibacterial properties of Ag nanoparticles arise from the attachment capability of these nanoparticles to the bacterial cell. This attachment results in the accumulation of envelope protein precursors, leading to protein denaturation, a decrease in proton motivation force, and finally, cell death.

## 3. Experimental Section

### 3.1. Materials

Waste polyethylene terephthalate (PET) bottles were collected from a local water packing company (Damavand Mineral Waters Company, Damavand City, Iran). Before dissolving in trifluoroacetic acid and preparing the solution for electrospinning, the labels of the PET bottles were removed, and then they were cleaned with detergents and dried. Trifluoroacetic acid (CF_3_COOH, TFA, 99%), ethanol (CH_3_CH_2_OH, 96%), and silver nitrate (AgNO_3_, ≥99.0%) were obtained from Merck (Darmstadt, Germany). Sodium hydroxide (NaOH, 98%) was prepared by Beijing Chemical Works (Beijing, China). Pure organic turmeric powder was purchased from a local grocery store. Microbes such as *Staphylococcus aureus* PTCC 1112, *Escherichia coli* PTCC 1338, and *Pseudomonas aeruginosa* PTCC 1074 were obtained from the microbiology laboratory, Islamic Azad University, Ahar branch, Iran. Nutrient agar as solid culture medium and nutrient broth as liquid culture medium were used for the growth of microorganisms. Double-distilled water was used in all experiments. All the reagents were used as received without further purification.

### 3.2. Fabrication of Electrospun PET Membrane

After removing the labels, the waste PET bottles were cut into squares of 10 × 10 mm^2^ with scissors and rinsed with water and ethanol and dried. Solution for electrospinning was prepared with a concentration of 10 wt.% (*w/v*) by dissolving 0.18 g of PET in 1 mL of trifluoroacetic acid. This solution was mixed constantly using a magnetic stirrer for 5 h to obtain a homogenous polymer solution.

**Figure 10:**
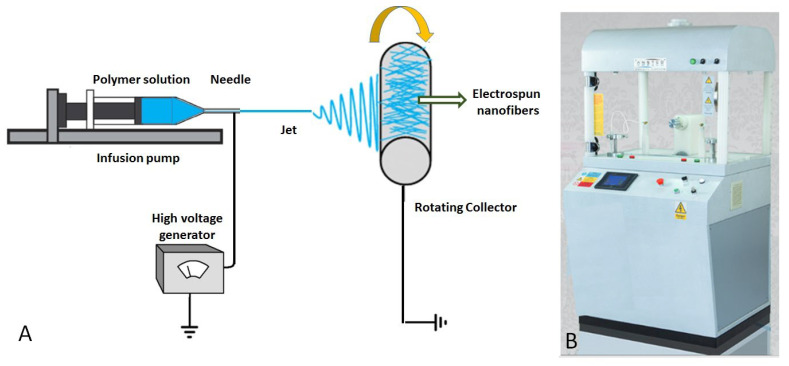
Schematic of the electrospinning setup (**A**) and electrospinning process (**B**).

Electrospinning experiments were performed according to our previous work [10]. Figure 10 shows the schematic of electrospinning process used to fabricate the nanofibers. The electrospinning machine consisted of a 2 mL syringe pump (SP 1000) with a stainless-steel needle (0.2 mm in diameter), a DC voltage supply in the kV range, and a collector. In this work, 20 kV voltage was used to induce the necessary charges on the solution and initiate the spinning. A rotating aluminum foil collector with the rotational speed of 250 rpm collected the electrospun nanofibrous membrane. The flow rate and the tip-to-collector distance were selected as 10 cm and 0.2 mL/h, respectively. The solution was electrospun for 1 h.

### 3.3. Modification of Electrospun PET Membrane

**Scheme 3:**
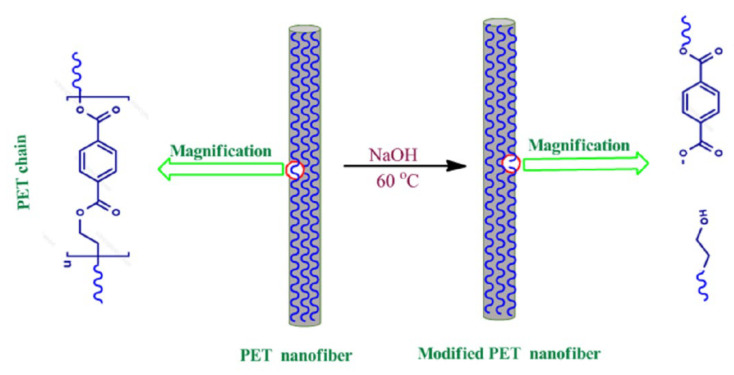
Schematic of surface modification reactions of PET nanofiber.

A total of 100 mg of nanofibrous membrane was taken in 10 mL of aqueous NaOH (0.2 g/L), and the mixture was heated at 60 °C for 5 min. Then, the membrane was washed with double-distilled water. The surface modification procedure is schematically shown in Scheme 3. During the alkaline hydrolysis of PET, the ester linkages were cleaved to form sodium terephthalate and ethylene glycol on the surface of nanofibers.

### 3.4. Preparation of the Curcumin Powder Extract

A total of 6.8 g of curcumin powder (organic turmeric) was added to 100 mL of double-distilled water, and the mixture was heated at 100 °C for 12 min. The obtained mixture was cooled and then filtered using Whatman filter paper (20–25 µm particle retention) and centrifuged for 10 min to obtain the extract for the green synthesis of Ag nanoparticles onto nanofibers of electrospun PET membrane.

### 3.5. Green Synthesis of Ag Nanoparticles onto Electrospun PET Nanofiber Surface

The green synthesis of Ag nanoparticles was achieved using antioxidants from turmeric extract [70]. Briefly, 100 mg of functionalized nanofibrous membrane was immersed in 10 mL of 10 mM Ag nitrate aqueous solution under moderate magnetic stirring at 25 °C. To ensure the adsorption of silver ion (Ag^+^) on the carboxylated chain PET of nanofibers, the obtained mixture was stirred at 25 °C for 4 h under N_2_ atmosphere. Next, 5 mL of prepared turmeric extract was added dropwise into the solution to initiate the reduction reaction. To complete the reduction reaction and green synthesis of Ag nanoparticles, the mixture was stirred using magnetic stirrer at 25 °C for 24 h. After stirring overnight, the color of the mixture changed from light yellow to dark-brown-reddish color. Also, the color of white nanofibrous membrane turned brown, and Ag-nanoparticle-decorated PET membranes were obtained. The obtained sample was washed twice with double-distilled water and dried at 25 °C.

### 3.6. Antibacterial Studies

Antibacterial activities of nanofibrous membranes were measured using Kirby–Bauer disc diffusion method. The microbes were cultured in a Muller–Hinton broth for 24 h at 37 °C. The 0.5 McFarland was visually used as a standard to adjust the turbidity of bacterial suspensions. The surface of preloaded Petri dishes with Mueller–Hinton agar was seeded with microbial suspensions using a sterile cotton swab. The bacterial suspensions were seeded on the surface of preloaded Petri dishes with Mueller–Hinton agar using a sterile cotton swab. The membranes were cut using a paper-punching machine in the form of discs with an approximate diameters of 6 mm. These disc-shaped samples were transferred onto the surface of the inoculated agar plate and incubated at 37 °C for 24 h. After incubation period, the zone of inhibition diameters was determined (measured by a ruler, sensitivity 1 mm). The equation (T − D)/2 was used to calculate the zone of inhibition size (mm), where T is the diameter of the clear zone around the test sample where microbes were not allowed to grow, and D is the diameter of the disc-shaped samples (6 mm). All the experiments were triplicated.

### 3.7. Effect of Nanofibrous Membranes on the Planktonic Cultures

To investigate the antimicrobial activity of nanofibrous membranes (*Staphylococcus aureus*, *Pseudomonas aeruginosa*, and *Escherichia coli*) in liquid medium (planktonic), the samples were cut into small discs with an approximate diameter of 6 mm and sterilized. Each disc-shaped sample was placed in one well of a sterile six-well plate. The microbial suspension was prepared in sterile physiological saline (0.9% sodium chloride solution), and its concentration (the approximate number of bacteria) was adjusted using 0.5 McFarland standard. Next, 0.2 cc of microbial suspension and 2 cc of nutrient broth were added over the deposited samples into the wells. The as-prepared six-well plates containing membrane samples, culture medium, and microbes were incubated at 37 °C for 24 h. To estimate the number of grown bacteria after the incubation time, the turbidity of the obtained microbial suspension (absorbance) was determined using UV-visible spectroscopy at 600 nm into a sterile quartz cuvette.

### 3.8. Investigation of Biofilm Formation

After the incubation time, nanofibrous membranes were separated from the microbial suspension prepared in the previous section and washed with sterile saline water to investigate their effect on microbial adhesion and biofilm formation. The samples were again placed in the new culture medium and incubated to continue the maturation of biofilm and the development of adhered microbial cells for another 24, 48, and 72 h at 37 °C. After the end of each incubation time, nonadherent microorganisms were removed from the colonized nanofibrous samples by washing, and the samples were placed in a sterile tube containing 1 cc sterile saline. The cells were separated from the biofilm formed on the surface of the samples using a vigorous vortex for 30 s and then sonication for 10 sec. The suspensions obtained from the separated cells were diluted, and various ten-fold serial dilutions were seeded on agar plates and incubated to obtain and quantify the number of viable cells expressed in colony-forming units (CFUs)/cc.

### 3.9. Characterization

The size and morphology of the electrospun membranes were investigated using HITACHI S-4160 field emission scanning electron microscope (FE-SEM). The morphology and the dispersion of Ag nanoparticles on the surface of nanofibers were illustrated using Zeiss Leo 906 (Carl Zeiss Inc., Oberkochen, Germany) transmission electron microscopy (TEM). Fourier transform infrared (FT-IR) spectra of membranes were recorded on a Tensor 27 FTIR spectrometer (Bruker Optik GmbH) using KBr discs in the region of 400–4000 cm^−1^. The crystalline phases were analyzed using X-ray diffractometer (Tongda TD-3700 model, Dandong, China) using copper Kα (λ = 1.541874 A) in the 2θ range of 10 to 85°. The step size and the step time selected were 0.02° and 1 s, respectively. UV-visible absorption spectrum of the nanofibrous membranes was measured in the range of 200–800 nm using a Cary 100 spectrophotometer.

## 4. Conclusions

It is very unlikely that the use and production of PET will be limited in the near future due to its positive properties for various applications. The high thermal and chemical stability of PET and its good resistance to biological atmospheric agents make it difficult to degrade and destroy in the environment. As a result, huge amounts of PET waste are being produced around the world, and this poses a global problem for the ecosystem. Hence, the recycling of PET is very significant to reduce the volume of its waste. In addition, the recycling process of PET and other plastics as a sustainable approach can produce value-added products from inexpensive resources by converting waste into valuable products. The current research successfully concluded the recycling and electrospinning of PET for the fabrication of an electrospun PET/Ag membrane. In summary, a PET solution was successfully converted to continuous, uniform, and bead-free nanofibers with smooth surfaces, and we obtained the electrospun membrane. Then, the electrospun PET membrane surface was modified with NaOH to decorate with Ag nanoparticles. A simple, low cost, and environmentally friendly technique was established to prepare Ag nanoparticle-decorated PET membranes. Ag nanoparticles were synthesized using the antioxidant constituents from turmeric extract, and the result confirmed the formation of Ag nanoparticles uniformly decorated on the PET nanofibers’ surface. The PET/Ag membrane showed good antimicrobial and antibiofilm activity against the *Staphylococcus aureus*, *Pseudomonas aeruginosa*, and *Escherichia coli* strains used in this study. It is concluded that the electrospun PET/Ag membrane could be used as a promising material for the eradication of these microbes, which are a common cause of local infections. The methodology presented here is a general and versatile method to prepare different kinds of electrospun polymer/metal using changing materials. The strong antimicrobial activity of this nanocomposite membrane makes it a promising material for various applications, including wound dressings, air filters, and water purification. However, further research is needed to optimize the production and performance of these materials for specific applications.

## Figures and Tables

**Table 1:** EDS results of electrospun PET membrane.

Element	Weight%	Atomic%
C	64.15	70.44
O	35.85	29.56

**Table 2:** EDS results of electrospun PET/Ag membrane.

Element	Weight%	Atomic%
C	59.74	67.28
O	38.42	32.49
Ag	1.84	0.23

## Data Availability

Data will be available on request.
